# *Legionella* pneumonia immediately after recovery from COVID‐19

**DOI:** 10.1002/ccr3.6090

**Published:** 2022-07-19

**Authors:** Daisuke Jingu, Hiroshi Takahashi, Satoshi Ubukata, Hiroshi Watanabe, Akira Horii

**Affiliations:** ^1^ Department of Respiratory Medicine Saka General Hospital Shiogama Japan; ^2^ Department of Internal Medicine Saka General Hospital Shiogama Japan

**Keywords:** COVID‐19, *Legionella* pneumonia, *Legionella pneumophila*, SARS‐CoV‐2, serogroup 5

## Abstract

We experienced a patient with *Legionella* pneumonia developing immediately after discharge from COVID‐19 recovery. Antibiotic treatment was successful. The source of *Legiolella* infection was proven to be bathtub water in this case. It is very important to accurately detect pathogens, particularly in the time of pandemics such as COVID‐19.

## Graphical abstract

Identification of the causative organism is extremely important in infectious disease. A subsequent infectious disease such as pneumonia immediately after recovery from COVID‐19 may pose a pitfall; legionellosis can be an important life‐threatening infectious disease. Patients should be carefully followed up after discharge.

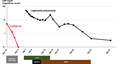

## 1 CLINICAL IMAGE

**FIGURE 1:**
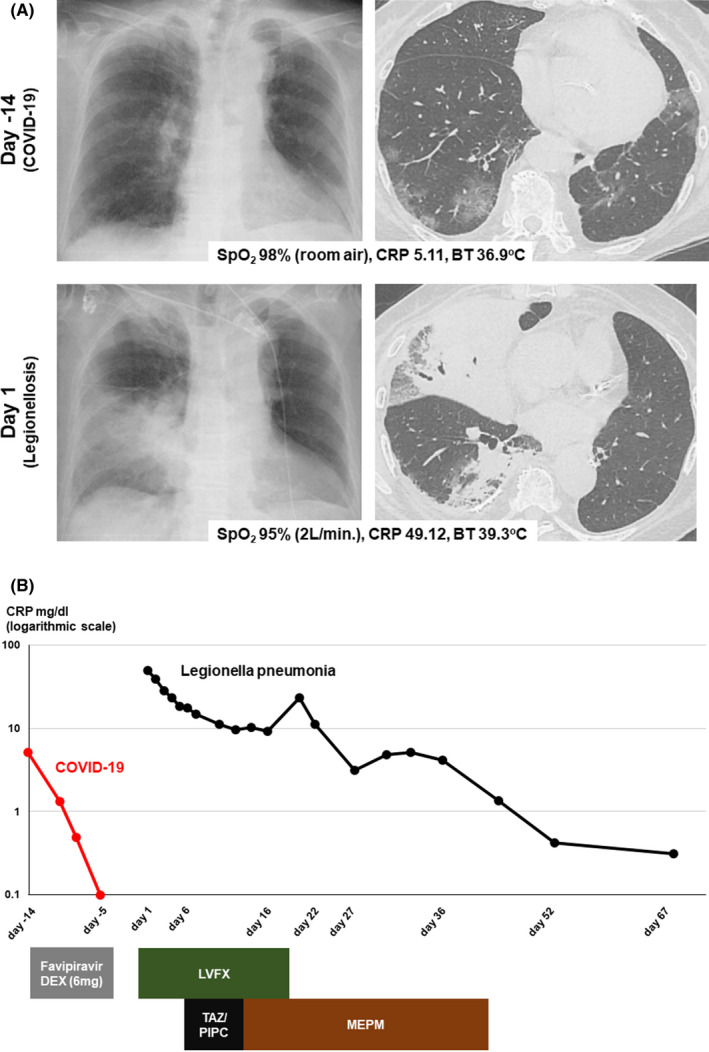
Clinical course of patient with images. (A) Images of plain X‐ray and CT scan on indicated date with percutaneous oxygen saturation (SpO_2_), CRP, and body temperature (BT). (B) Clinical course of CRP with treatments. This patient was hospitalized with COVID‐19 between Day −14 and Day −5, then, re‐hospitalized on Day 1 with Legionnaire's disease. DEX, dexamethasone; LVFX, levofloxacin; TAZ/PIPC, tazobactam/piperacillin; MEPM, meropenem.

**FIGURE 2:**
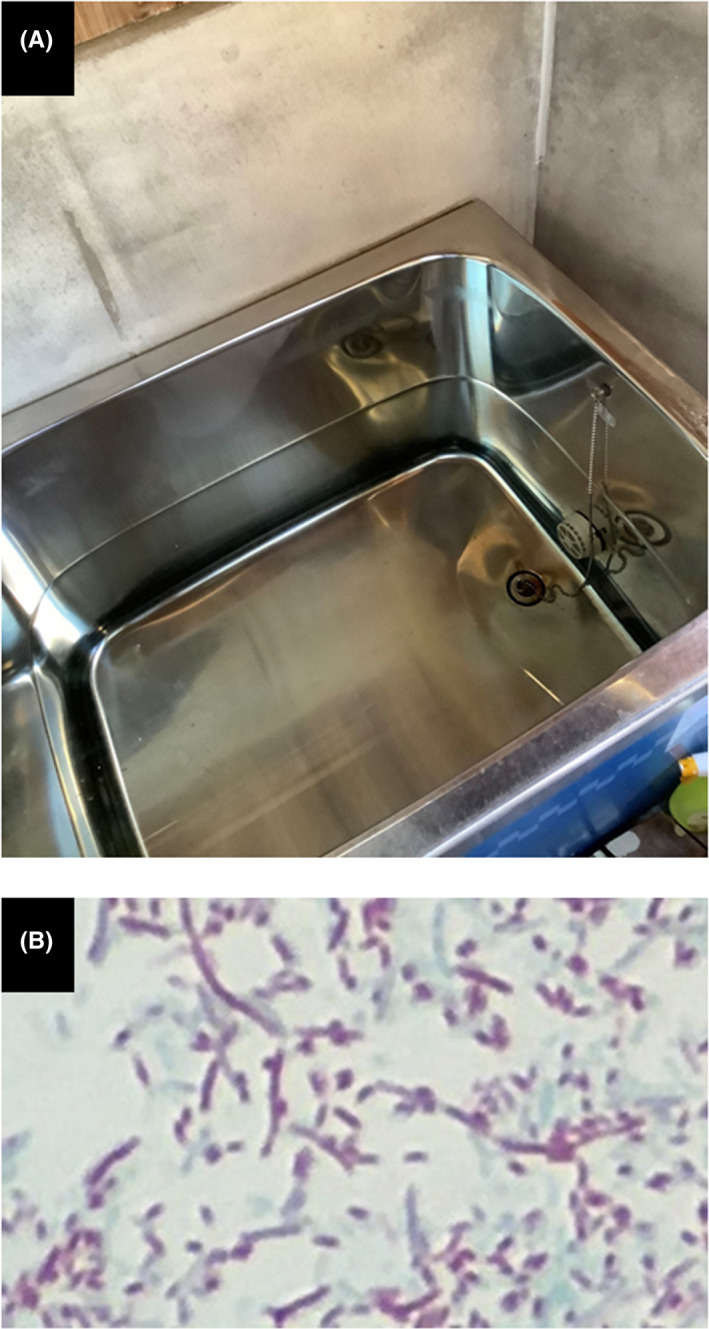
(A) Bathtub filled with reused water in this patient's home. (B) Gimenez staining disclosed *Legionella pneumophila* in both patient's sputum and bathtub water. Staining results from bathtub water are shown.

An 83‐year‐old woman visited our emergency department with consciousness disorder, high fever, and hypoxemia 5 days after healing and discharge from COVID‐19. Examination data on Day 1 revealed high inflammatory responses. Chest X‐ray and CT (Figure 1A) revealed features of severe pneumonia which are obviously distinct from those at COVID‐19. The SARS‐CoV‐2 antigen was negative, but urinary *Legionella* antigen was positive, and *Legionella pneumophila* serogroup 5 was detected from her sputum. *Legionella* pneumonia was diagnosed. Levofloxacin was effective for the *Legionella* pneumonia, but secondary bacterial pneumonia developed; other antibiotic therapy was successful. She was transferred to a long‐term hospital on Day 68 (Figure 1B). *Legionella pneumophila* was detected in her daily reused bathtub water (Figure 2); this very common Japanese lifestyle in Japan can be a hotbed of *Legionella* infection. Increasing focus on Legionnaires' disease is needed in association with relaxation of lockdown as the COVID‐19 pandemic recedes.^1^ Legionellosis after influenza is reported,^2^ but this is the first case of *Legionella* pneumonia after COVID‐19 infection. Patients should be followed up for subsequent infections after discharge.

## AUTHOR CONTRIBUTIONS

DJ, HT, SU, and HW substantially contributed to the diagnosis and clinical care of the patient. DJ wrote the draft of the manuscript, and DJ and AH critically revised it for important intellectual content.

## FUNDING INFORMATION

None.

## CONFLICT OF INTEREST

None declared.

## CONSENT

Written informed consent was obtained from the patient to publish this report in accordance with the journal's patient consent policy, and the study was approved by the Ethics Committee in Saka General Hospital (No. 21–9‐21).

## Data Availability

No data are available in this case report.
